# TMPRSS2 Serves as a Prognostic Biomarker and Correlated With Immune Infiltrates in Breast Invasive Cancer and Lung Adenocarcinoma

**DOI:** 10.3389/fmolb.2022.647826

**Published:** 2022-04-26

**Authors:** Xinhua Xiao, Huizhuang Shan, Yangyang Niu, Peihong Wang, Donghe Li, Yuyin Zhang, Jiayi Wang, Yingli Wu, Hua Jiang

**Affiliations:** ^1^ Department of Hematology and Oncology, Guangzhou Women and Children’s Medical Center, Guangzhou Medical University, Guangdong, China; ^2^ Laboratory Medicine, Guangdong Provincial People’s Hospital, Guangdong Academy of Medical Sciences, Guangzhou, China; ^3^ Department of Nephrology, Shanghai Tongji Hospital, Tongji University School of Medicine, Shanghai, China; ^4^ State Key Laboratory of Medical Genomics, Collaborative Innovation Center of Hematology, National Research Center for Translational Medicine, Ruijin Hospital, Shanghai Institute of Hematology, Shanghai Jiao Tong University School of Medicine, Shanghai, China; ^5^ Key Laboratory of Cell Differentiation and Apoptosis of the Chinese Ministry of Education, Shanghai Tong Ren Hospital/Faculty of Basic Medicine, Hongqiao International Institute of Medicine, Chemical Biology Division of Shanghai Universities E-Institutes, Shanghai Jiao Tong University School of Medicine, Shanghai, China

**Keywords:** tmprss, tumor immune infiltrating cells, prognosis, breast invasive cancer, lung adenocarcinoma

## Abstract

TMPRSS2 is a transmembrane serine protease and plays a pivotal role in coronavirus disease 2019 (COVID-19). However, the correlation of TMPRSS2 with prognosis and immune infiltration in tumors has not yet been explored. Here, we analyzed the expression of TMPRSS2 in Oncomine and TIMER databases, the correlation between TMPRSS2 and overall survival in the PrognoScan, Kaplan-Meier plotter, and GEPIA databases. The association between TMPRSS2 and immune infiltration levels was investigated in the TIMER database. In addition, the prognosis of TMPRSS2 related to immune cells in cancers was analyzed. Quantitative real-time PCR (qRT-PCR) confirmed that TMPRSS2 was upregulated in lung adenocarcinoma (LUAD) and downregulated in breast invasive carcinoma (BRCA). We demonstrated that high TMPRSS2 expression was associated with favorable prognosis in LUAD, but it was associated with poor prognosis in BRCA. Interestingly, we found that TMPRSS2 expression was significantly correlated with immune infiltration of B cells, CD4^+^ T cells, macrophages, and dendritic cells in LUAD, and it was positively correlated with the infiltrating levels of CD8^+^ T cells, CD4^+^ T cells, neutrophils, and dendric cells in BRCA. Consistent with the prognosis of TMPRSS2 in LUAD and BRCA, the high expression level of TMPRSS2 has a favorable prognosis in enriched immune cells such as B cells, macrophages, and CD4^+^ T cells in LUAD, and it has a poor prognosis in CD4^+^ T cells and CD8^+^ T cells in BRCA. In conclusion, our results indicate that the prognosis of TMPRSS2 in LUAD and BRCA is significantly correlated with immune cells infiltration. Our study comprehensively revealed the relationship between the prognosis of TMPRSS2 in pan-cancers and tumor immunity.

## Introduction

Lung cancer is currently the leading cause of cancer death; despite therapeutic advances over the last several decades, the 5 year overall survival remains only 19% (Siegel et al., 2020). Lung adenocarcinoma (LUAD) belonging to the subtype of non-small-cell lung carcinoma (NSCLC) is characterized by the most common histological subtype of lung cancer (Travis et al., 2011), which comprises more than 40% of all lung cancers (Meza et al., 2015). Patients with LUAD have biological heterogeneity and harbor several gene activation mutations such as EGFR and KRAS, making the therapy of LUAD more formidable (Skoulidis et al., 2015). However, significant and effective anti-cancer strategies in immunotherapy made the treatment of LUAD more promising; that is, anti-CTLA4 and anti-PD1 antibodies were widely applied to improve chemotherapy (Topalian et al., 2011). Therefore, it is essential to explore the relationship between tumor biomarkers and immune cells.

Breast cancer is the most common cancer in women and accounts for 30% of female cancers (Siegel et al., 2020). Breast invasive carcinoma (BRCA) is a metastatic, malignant, and poor prognosis type of breast cancer that invades the basement membrane layers to extend into the surrounding stroma. The malignant progression of breast cancer is affected by multiple microenvironment components, such as immune cells, adipocytes, fibroblasts, cytokines, and chemokines (Goff and Danforth, 2021). Immune cell infiltration was recognized as a novel biological marker in breast cancer (Toss and Cristofanilli, 2015). Moreover, the prognosis of breast cancer was related to tumor-infiltrating immune cells (Yamaguchi et al., 2012; Mittal et al., 2014). Thus, it is meaningful to further investigate the function of immune cells in breast cancer.

Transmembrane serine protease 2 (TMPRSS2) is a transmembrane serine protease predominantly expressed in epithelial cells of the prostate gland and classified as a type II transmembrane serine protease (TTSP) (Lin et al., 1999; Lucas et al., 2008). The pivotal role of TMPRSS2 in the progression of prostate cancer is widely studied. TMPRSS2 interacts with the tumor microenvironment and promotes prostate cancer cell invasion, tumor growth, and metastasis (Lucas et al., 2014; Ko et al., 2015). In addition, TMPRSS2 is also expressed in the aerodigestive tract, facilitating the entry of coronavirus particles into cells by cleaving the spike protein and making people more susceptible to infection of SARS-CoV-2 (Hoffmann et al., 2020; Walls et al., 2020; Wrapp et al., 2020). However, the function of TMPRSS2 in the progression of tumors and the relationship between the tumors and tumor immunity remains unknown.

In the current study, we systematically analyzed and further detected the expression of TMPRSS2 in tumors and adjacent tissues through qRT-PCR and then analyzed prognosis by several databases such as Oncomine, TIMER, PrognoScan, GEPIA, and Kaplan-Meier plotter. Additionally, we also analyzed the relationship between TMPRSS2 and immune cells in the tumor microenvironment. Our results uncovered the key function of TMPRSS2 in tumor prognosis and provided an insight into the prognosis affected by tumor immune cells.

## Materials and Methods

### PrognoScan Database Analysis

The PrognoScan database (http://www.abren.net/PrognoScan/) (Mizuno et al., 2009) was applied to analyze the relationships between TMPRSS2 expression and prognosis in different types of cancers. We searched overall survival (OS), relapse-free survival, and distant metastasis-free survival of TMPRSS2 in cancers from cancer microarray datasets. The threshold was Cox *p*-value < 0.05.

### Oncomine Database Analysis

TMPRSS2 expression in various types of cancers was identified in the Oncomine database (https://www.oncomine.org/resource/main. html) (Rhodes et al., 2007). The threshold was defined as follows: *p*-value of 0.01, fold change of 1.5, and a top 10% of gene ranking.

### TIMER Database Analysis

TIMER is a comprehensive database for analysis of the gene expression in different tumors and immune cells infiltrations in various types of cancers (https://cistrome.shinyapps.io/timer/) (Li et al., 2017). We analyzed the TMPRSS2 expression between tumor and adjacent normal tissues across all TCGA tumors in the TIMER database and the differential expression was shown with statistical significance. Moreover, we analyzed the correlation of TMPRSS2 expression with immune cells infiltration, including B cells, CD4^+^ T cells, CD8^+^ T cells, macrophages, neutrophils, and dendritic cells in the TIMER. TMPRSS2 expression levels against tumor purity are displayed on the left-most panel (Aran et al., 2015). The gene markers of tumor-infiltrating immune cells including CD8^+^ T cells, B cells, macrophages, neutrophils, and dendritic cells were reported in some studies (Sousa and Määttä, 2016; Danaher et al., 2017). The correlation of TMPRSS2 expression with immune cell infiltration in cancers was analyzed based on the gene markers. The *x*-axis represents the TMPRSS2 gene and the *y*-axis represents immune cell biomarkers. The gene expression level is displayed with log2 RSEM.

### Kaplan-Meier Plotter Database Analysis

Sources for Kaplan-Meier plotter databases include GEO, EGA, and TCGA databases. The Kaplan-Meier plotter is capable of assessing the effect of 54k genes on survival in 21 cancer types including 6,234 breast, 2,190 ovarian, 3,452 lung, and 1,440 gastric cancers. The relationships between TMPRSS2 expression level and the prognosis in breast, ovarian, lung, and gastric cancer were analyzed with the Kaplan-Meier plotter (http://kmplot.com/analysis/) (Györffy et al., 2010). The hazard ratio (HR) with 95% confidence intervals and log-rank *p*-value were calculated.

### GEPIA

Gene Expression Profiling Interactive Analysis (GEPIA) is a web-based tool that conveniently mines the survival of patients based on TCGA and GTEx data (Tang et al., 2017). The survival of TMPRSS2 expression in cancers was also obtained from the online database GEPIA (http://gepia.cancer-pku.cn/index.html).

### Statistical Analysis

The results analyzed in Oncomine are displayed with *p*-values, fold changes, and ranks. The different expression of TMPRSS2 in cancers analyzed by the TIMER database was exhibited with *p*-values. Survival curves of TMPRSS2 in various cancers were obtained from the GEPIA, PrognoScan, and Kaplan-Meier plots database and the results from the above database were displayed with HR and *P* or Cox *p*-values from a log-rank test. The results for the correlation between TMPRSS2 expression and immune infiltration level in various cancer types were generated and displayed with Spearman’s correlation and statistical significance. *p*-values < 0.05 was considered statistically significant.

## Results

### The mRNA Expression Levels of TMPRSS2 in Cancers

**FIGURE 1:**
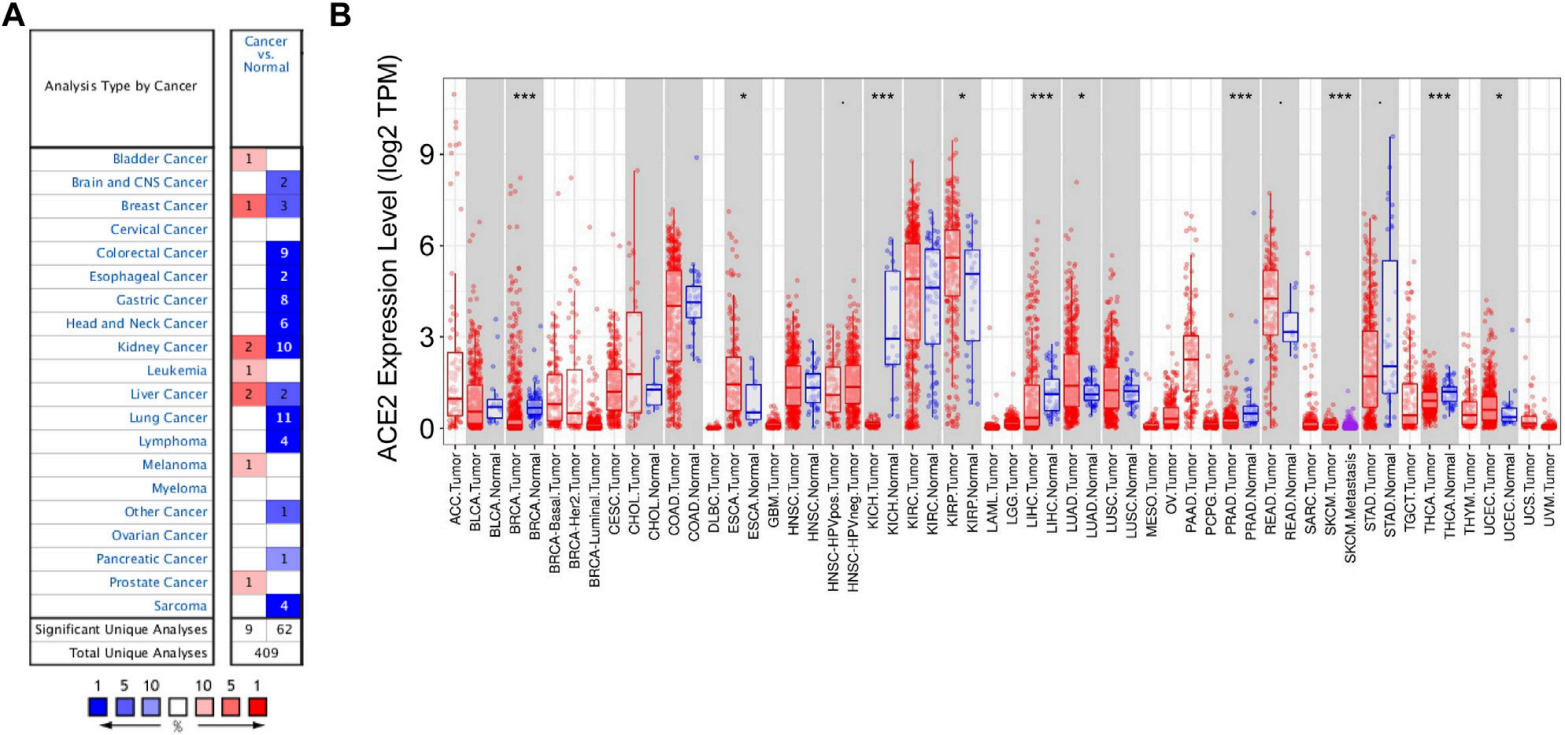
Expression levels of TMPRSS2 in different types of cancers. **(A)** Relative expression of TMPRSS2 in different cancers compared with normal tissues in the Oncomine database. **(B)** TMPRSS2 expression levels in different tumor types from the TCGA databases were detected by TIMER (*p < 0.05, **p < 0.01, ***p < 0.001).

To explore TMPRSS2 expression in various cancers, we analyzed the mRNA expression levels of TMPRSS2 through the Oncomine database. The results showed that TMPRSS2 was highly expressed in bladder cancer, breast cancer, kidney cancer, leukemia, liver cancer, melanoma, and prostate cancer. In contrast, TMPRSS2 level was downregulated in the brain and CNS cancer, breast cancer, colorectal cancer, esophageal cancer, head and neck cancer, lung cancer, lymphoma, other cancer (mixed germ cell tumor, embryonal carcinoma, yolk sac tumor, testicular embryonal carcinoma, seminoma, testicular yolk sac tumor, uterine corpus leiomyoma, malignant fibrous histiocytoma, testicular seminoma), pancreatic cancer, and sarcoma (Figure 1A).

Furthermore, in order to assess TMPRSS2 expression levels across tissues, we analyzed the expression of TMPRSS2 in the tumor and adjacent normal tissues based on the RNA-seq data of the various malignancies in the TCGA database. Compared with the normal tissues, the expression of TMPRSS2 was higher in ESCA (esophageal carcinoma), KIRP (kidney renal papillary cell carcinoma), LUAD (lung adenocarcinoma), READ (rectum adenocarcinoma), and UCEC (uterine corpus endometrial carcinoma) (Figure 1B). However, the expression of TMPRSS2 was lower in BRCA (breast invasive carcinoma), KICH (kidney chromophobe), LIHC (liver hepatocellular carcinoma), PRAD (prostate adenocarcinoma), STAD (stomach adenocarcinoma), and THCA (thyroid carcinoma) (Figure 1B).

### The Prognosis of TMPRSS2 in Various Cancers

**FIGURE 2:**
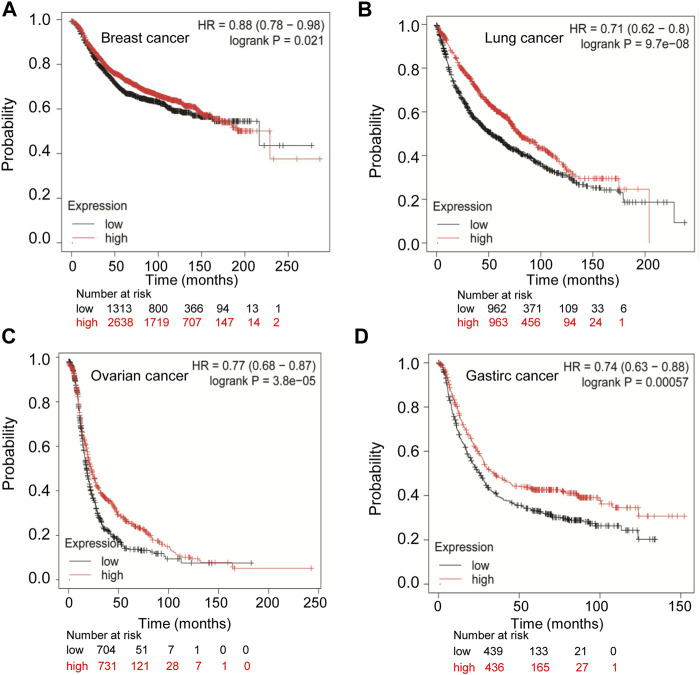
Comparison of Kaplan-Meier survival curves of highly expressed and lowly expressed TMPRSS2 in different cancers. **(A)** High expression of TMPRSS2 in breast cancer (*n* = 3951) had a poor prognosis analyzed by the Kaplan-Meier plotter database. High expression of TMPRSS2 had favorable prognosis in lung cancer (*n* = 1925) **(B)**, ovarian cancer (*n* = 1435) **(C),** and gastric cancer (*n* = 875) **(D)** analyzed by Kaplan-Meier plotter database.

Since the expression of TMPRSS2 in cancers is diverse, we wondered whether TMPRSS2 is associated with tumor prognosis. We first evaluated the relationship between the expression of TMPRSS2 and overall survival rate in different tumors by PrognoScan (Supplementary Table 1). In order to further analyze the prognosis of TMPRSS2 in cancers, Kaplan-Meier plotter databases and GEPIA databases were utilized. Obviously, the high expression of TMPRSS2 showed a poor prognosis in breast cancer (OS HR = 0.88, 95% CI = 0.78 to 0.98, *p* = 0.021) (Figure 2A), while it showed favorable prognosis in lung cancer, ovarian cancer, and gastric cancer (OS HR = 0.71, 95% CI = 0.62 to 0.8, *p* = 9.7e-08; OS HR = 0.77, 95% CI = 0.68 to 0.87, *p* = 3.8e-05; OS HR = 0.74, 95% CI = 0.63 to 0.88, *p* = 0.0057 respectively) (Figures 2B–D). Furthermore, the high expression of TMPRSS2 was associated with good prognosis in KIRP (OS HR = 0.49, 95% CI = 0.27 to 0.9, *p* = 0.019), LUAD (OS HR = 0.58, 95% CI = 0.44 to 0.78, 0.00023), UCEC (OS HR = 0.45, 95% CI = 0.31 to 0.7, *p* = 0.00018), LIHC (OS HR = 0.67, 95% CI = 0.46 to 0.97, *p* = 0.032) and STAD (OS HR = 0.66, 95% CI = 0.47 to 0.94, *p* = 0.022) (Supplementary Figure S1). However, the high expression of TMPRSS2 showed a poor prognosis in READ (OS HR = 2.2, 95% CI = 1 to 4.84, *p* = 0.043), breast cancer (OS HR = 1.59, *p* = 0.0047), and BRCA (OS HR = 1.5, *p* = 0.015) (Supplementary Figures 1C, H, I). The TMPRSS2 expression had no significant correlations with the prognosis of THCA, ESCA, PRAD, and KICH (Supplementary Figures 1G, J, K, L). These results suggested that the prognosis of TMPRSS2 in various cancers was different and the prognostic value depended on the type of cancer.

### The Expression Level of TMPRSS2 is Correlated With Tumor Immune infiltration

**FIGURE 3:**
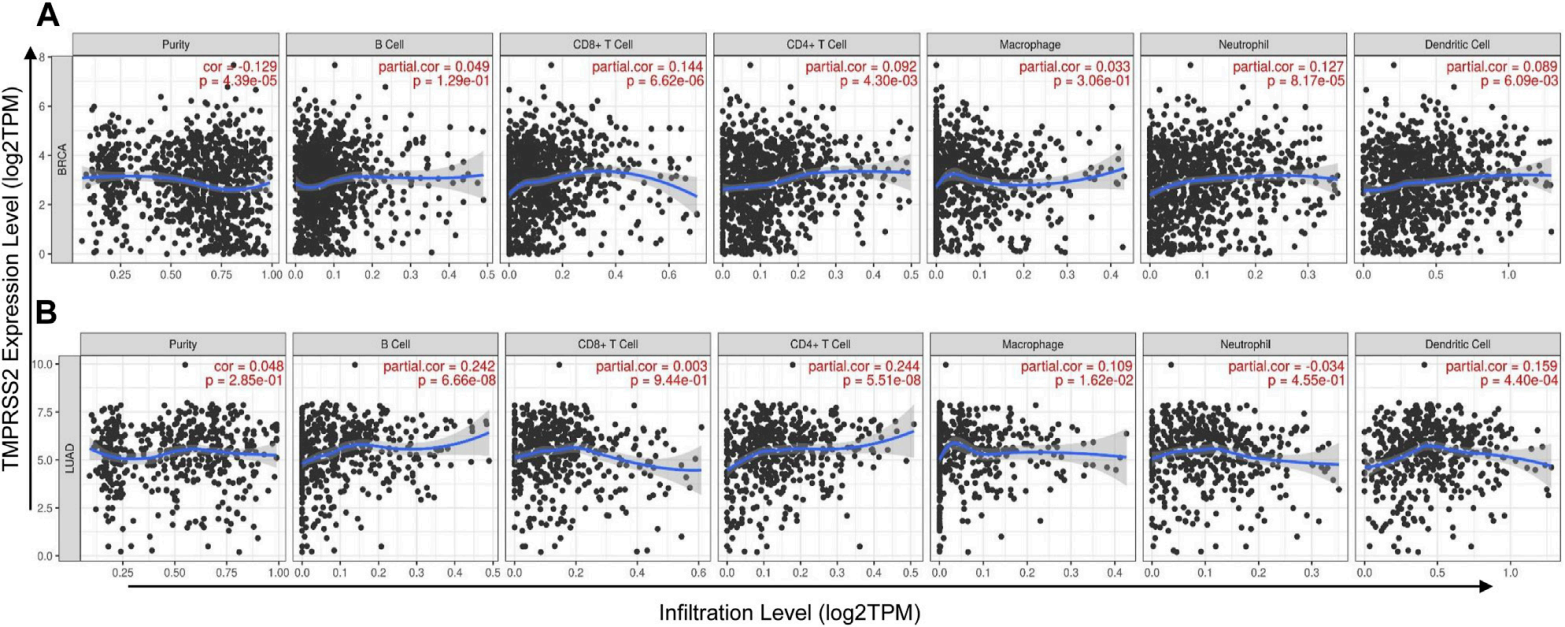
Correlation of TMPRSS2 expression with immune cells infiltration level in BRCA (breast invasive carcinoma) and LUAD (lung adenocarcinoma) were detected by TIMER. **(A)** TMPRSS2 expression is positively correlated with infiltrating levels of CD8^+^ T cells, CD4^+^ T cells, neutrophils, and dendritic cells in BRCA. **(B)** TMPRSS2 expression is positively correlated with infiltrating levels of B cells, CD4^+^ T cells, macrophages, and dendritic cells in LUAD.

Tumor immune infiltration was an independent prognostic factor for survival and was associated with the prognosis of malignant tumor (Loi et al., 2013; Glajcar et al., 2018), demonstrating that tumor immune infiltration may serve as a promising target for immune-based therapy and cancer prevention (Thompson et al., 2016). Therefore, we investigated whether the expression levels of TMPRSS2 in different types of cancers were correlated with immune infiltration levels. Tumors related to clinical prognosis were focused and used to further analyze the correlations between TMPRSS2 expression levels and immune infiltration in the TIMER database. The results revealed that TMPRSS2 expression was positively correlated with the level of immune infiltration of CD8^+^ T cells (r = 0.144, *p* < 0.001), CD4^+^ T cells (r = 0.092, *p* < 0.01), neutrophils (r = 0.127, *p* < 0.001), and dendric cells (r = 0.089, *p* < 0.01) in BRCA (Figure 3A). Similarly, there were positive correlations with infiltrating levels of B cells (r = 0.242, *p* < 0.001), CD4^+^ T cells (r = 0.244, *p* < 0.001), macrophages (r = 0.109, *p* < 0.05), and dendric cells (r = 0.159, *p* < 0.001) in LUAD (Figure 3B). However, TMPRSS2 expression has very weak and positive correlations with infiltrating levels of B cells, CD8^+^ T cells, CD4^+^ T cells, macrophages, neutrophils, and dendric cells in KIRP, READ, UCEC, LIHC, and STAD (Supplementary Figures S2). These results indicated that TMPRSS2 plays a critical role in immune cell infiltration in BRCA and LUAD.

### Correlation of TMPRSS2 Expression With Immune Cell Type Markers

To further confirm the relationships between the expression of TMPRSS2 and the various immune infiltrating cells. We analyzed the typical markers of B cells, CD8^+^ T cells, CD4^+^ T cells, macrophages, neutrophils, and dendric cells associated with BRCA and LUAD by the TIMER database.

**FIGURE 4:**
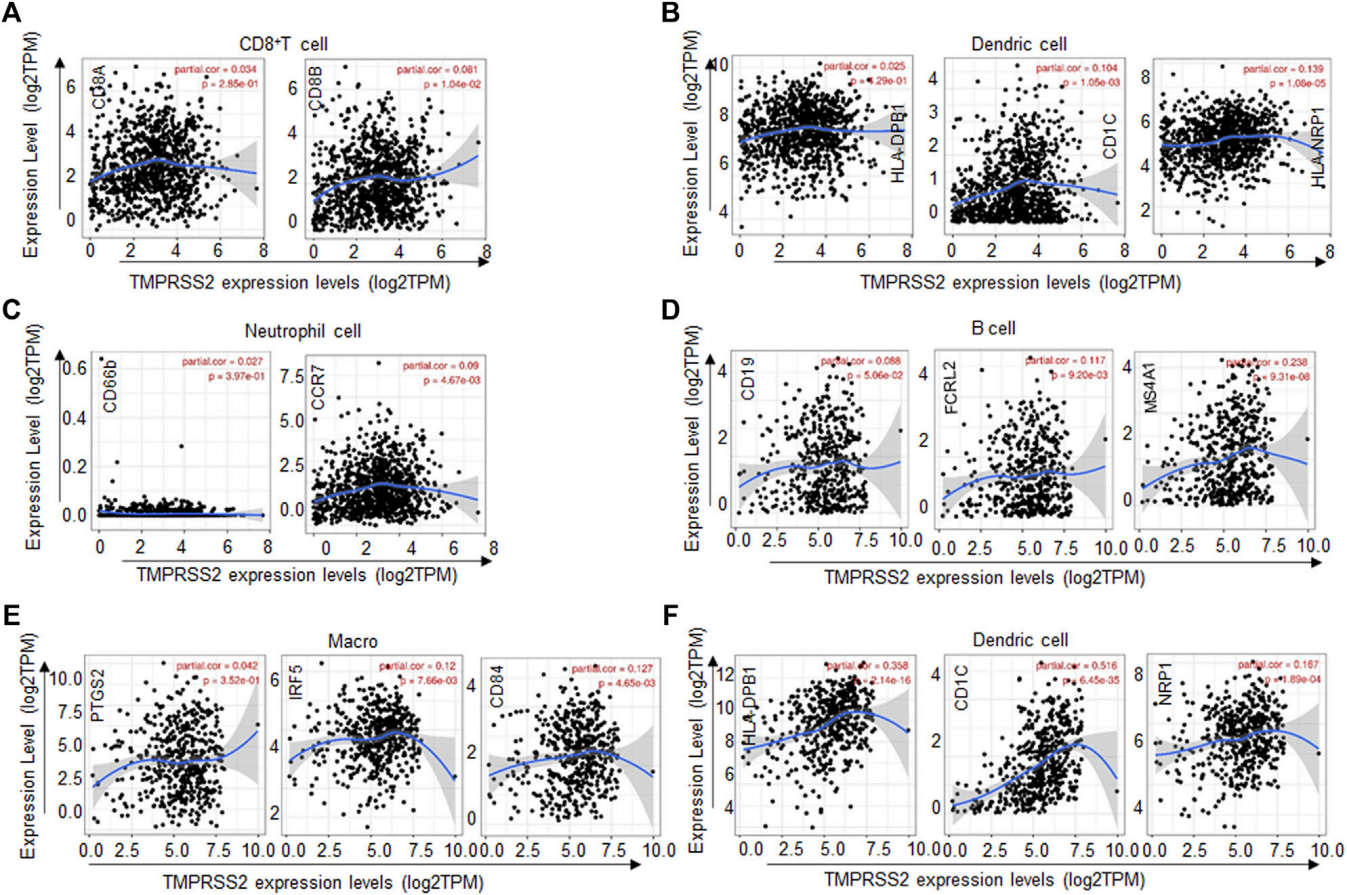
TMPRSS2 expression correlated with the immune cell type markers in BRCA (breast invasive carcinoma) and LUAD (lung adenocarcinoma). The correlation of TMPRSS2 expression with gene markers of CD8^+^ T cells **(A)**, dendric cells **(B),** and neutrophil cells **(C)** in BRCA. The correlation of TMPRSS2 expression with gene markers of B cells **(D)**, macrophages **(E),** and dendric cells **(F)** in LUAD. Gene markers of CD8^+^ T cells including CD8A and CD8B, gene markers of B cells including CD19, FCRL2, and MS4A1, gene markers of dendric cells including HLA-DPB1, CD1C, and HLA-NRP1, gene markers of neutrophil including CD66b and CCR7, gene markers of macrophages including PTGS2, IRF5, and CD84.

As described above, TMPRSS2 in BRCA was positively correlated with CD8^+^ T cells, CD4^+^ T cells, neutrophils, and dendric cells. In addition, CD8^+^ T-cell marker CD8B was positively correlated with TMPRSS2 expression (Figure 4A), dendric cell markers CD1C, and HLA-NRP1 were positively correlated with TMPRSS2 expression (Figure 4B) and neutrophils cell marker CCR7 was positively correlated with TMPRSS2 expression (Figure 4C). Similarly, the TMPRSS2 expression in LUAD was positively correlated with FCRL2 and MS4A1 in B cells (Figure 4D), IRF5, and CD84 in macrophages (Figure 4E) and HLA-DPB1, CD1C, and NRP1 in dendric cells (Figure 4F). These results further validated that TMPRSS2 is relevant to immune infiltrating cells in BRCA and LUAD.

### Prognostic Analysis of TMPRSS2 Expression in Tumor Based on Immune Cells

We have confirmed that the expression of TMPRSS2 was not only correlated with the prognosis of LUAD and BRCA but also correlated with the immune infiltrating cells in these tumors. Tumor immune infiltrating cells serve as an independent prognostic factor for survival and have a potent anti-tumor function in lung cancer and breast cancer (Thompson et al., 2016; Wang et al., 2019). Therefore, we speculated that the prognosis of lung cancer and breast cancer correlated with TMPRSS2 expression may be affected by the tumor immune infiltrating cells.

**FIGURE 5:**
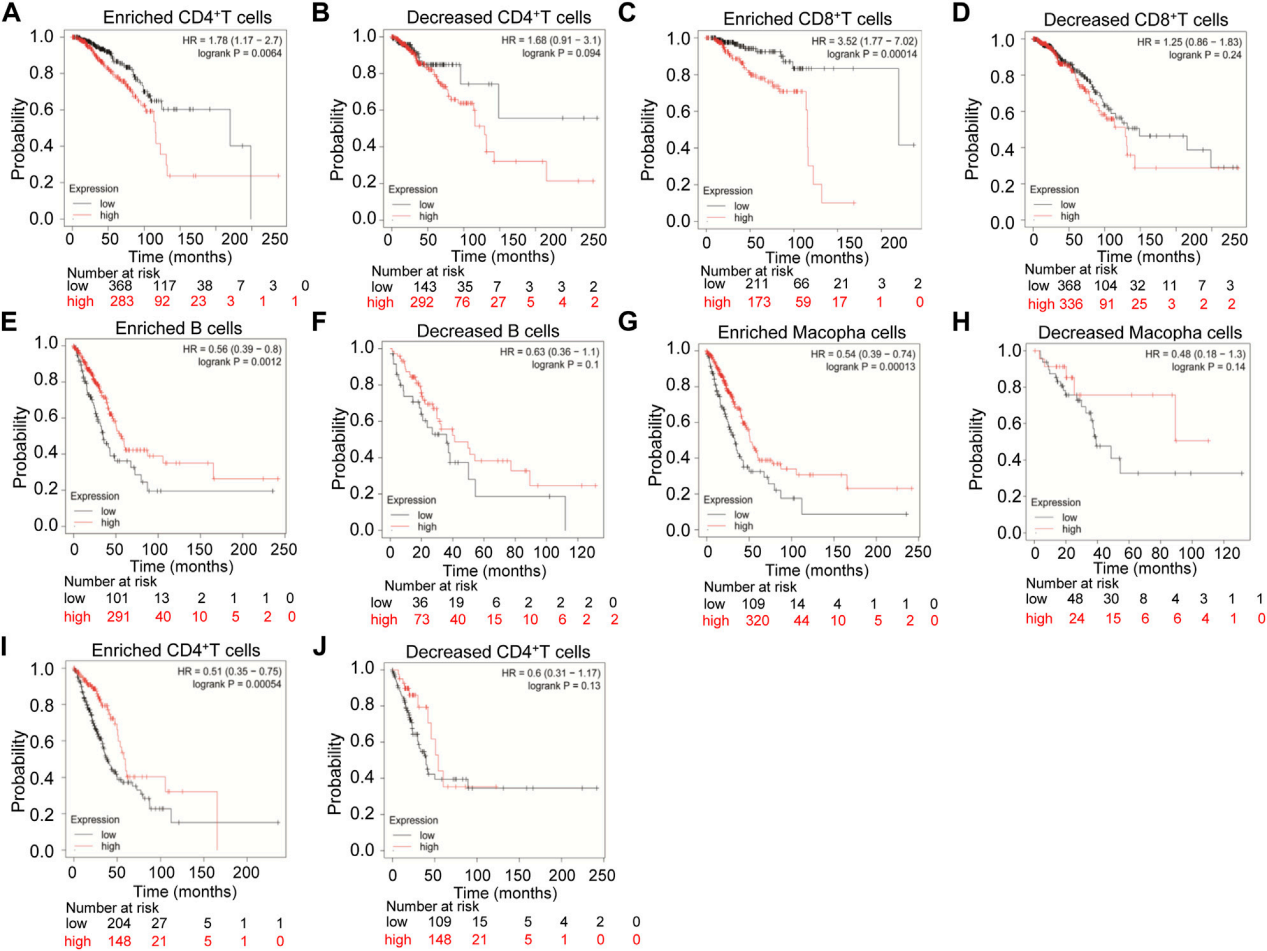
Kaplan-Meier survival curves compare the high and low expression levels of TMPRSS2 in breast cancer and LUAD based on immune cells. The prognosis of various expression of TMPRSS2 correlated with immune cells in breast cancer **(A–D)**, and LUAD **(E–J)**.

Therefore, we examined the prognosis of various immune infiltrating cells in breast cancer and lung cancer based on the TMPRSS2 expression level by the Kaplan-Meier plotter database. The results showed that high expression of TMPRSS2 in breast cancer has a poor prognosis in enriched CD4^+^ T cells (HR = 1.78 *p* < 0.01) and enriched CD8^+^ T cells (HR = 3.52 *p* < 0.001) (Figures 5A, C). But in LUAD, the high expression levels of TMPRSS2 has a better prognosis in enriched B cells (HR = 0.56, *p* < 0.01), enriched macrophage cells (HR = 0.54 *p* < 0.001), and enriched CD4^+^ T cells (HR = 0.51 *p* < 0.001), respectively (Figures 5E, G, I). Furthermore, there was no significant correlation between TMPRSS2 expression and the prognosis of breast cancer in decreased CD4^+^ T cells (*p* = 0.094) and decreased CD8^+^ T cells (*p* = 0.24) (Figures 5B, D). Similarly, there was no significant correlation between the expression of TMPRSS2 and the prognosis of LUAD in decreased B cells (*p* = 0.1), in decreased macrophage cells (*p* = 0.14) and decreased CD4^+^ T cells (*p* = 0.13) respectively (Figures 5F, H, I). Interestingly, the prognosis of breast cancer and LUAD affected by the TMPRSS2 expression is consistent with the respective prognosis based on immune cells (Figure 2; Supplementary Figures S2; Figure 5). These findings indicated that the prognosis of LUAD and breast cancer affected by the high expression of TMPRSS2 may be partially influenced by the immune infiltrating cells.

## Discussion

A novel severe acute respiratory syndrome coronavirus (SARS-CoV-2) led to the outbreak of coronavirus disease 2019 (COVID-19), causing infection and death in millions of people (Wu et al., 2020; Zhou et al., 2020). In the process of the infection of SARS-CoV-2, angiotensin-converting enzyme 2 (ACE2) enables the attachment of the virus and the host cell (Letko et al., 2020; Walls et al., 2020). Upon attaching the host cell membrane, SARS-CoV-2 binds with the human TMPRSS2 and then TMPRSS2 cleaved hemagglutinin and activated the entry of the virus into the host cell (Hoffmann et al., 2020; Matsuyama et al., 2020), which emphasized the essential function of TMPRSS2 in the pathogenesis of SARS-CoV-2. In addition, to involve in the infection of SARS-CoV-2, TMPRSS2 is also closely related to the carcinogenesis of prostate cancer and highly expressed in prostate cancer, and dominated the progression of prostate cancer (Lin et al., 1999; Stopsack et al., 2020). However, the role of TMPRSS2 in other cancers remains unknown.

In this study, we analyzed the mRNA expression of TMPRSS2 in various cancers in Oncomine and TIMER databases. We found TMPRSS2 expression varies in cancers (Figure 1A). Compared with adjacent normal tissues, the expression of TMPRSS2 was higher in ESCA, KIRP, LUAD, READ, and UCEC and was lower in BRCA, KICH, LIHC, PRAD, STAD, and THCA (Figure 1B). Consistent with the results analyzed from the database, we examined the mRNA expression of the TMPRSS2 gene in human cancer cell lines or tumor tissues of LUAD and BRCA and confirmed that compared with normal cells or tissues, TMADSS2 is highly expressed in LUAD and lowly expressed in BRCA (Supplementary Figures S3A–C). Abnormal TMPRSS2 expression indicates that TMPRSS2 may play a role in the progression of these cancers. Therefore, we analyzed the prognosis of TMPRSS2 in abnormally expressed cancers using the Kaplan-Meier plotter databases and GEPIA databases, and we found that increased TMPRSS2 expression was correlated with a better prognosis in KIRP, LUAD, UCEC, LIHC, and STAD (Supplementary Figures S1A–F). However, the increased expression of TMPRSS2 showed a poor prognosis in READ, breast cancer, and BRCA (Supplementary Figures S1C, H, I). The prognosis of the cancers analyzed by Kaplan-Meier plotter databases and GEPIA databases indicating that TMPRSS2 may serve as an independent risk factor to evaluate the prognosis of KIRP, LUAD, UCEC, LIHC, STAD, READ and breast cancer, or BRCA.

Tumor microenvironment (TME) such as the immune compartment, stromal cells, extracellular matrix, and metabolism play a key factor in the genesis, progression, and treatment of tumor (Burgos-Panadero et al., 2019). In particular, the immune cells in the tumor microenvironment were correlated with the tumor progression and tumor therapy effect (Lucas et al., 2014; Chen and Mellman, 2017). Interestingly, we found that TMPRSS2 expression was correlated with diverse immune cell infiltration levels in cancers, especially in LUAD and BRCA. Analysis from the TIMER database demonstrated that TMPRSS2 expression was positively correlated with immune infiltration levels of CD8^+^ T cells, CD4^+^ T cells, neutrophils, and dendric cells in BRCA (Figure 3A). In addition, there were positive relationships between TMPRSS2 expression and infiltration levels of B cells, CD4^+^ T cells, macrophages, and dendric cells in LUAD (Figure 3B). Furthermore, the increased expression of TMPRSS2 was positively correlated with the expression of dendric cells markers, CD8^+^ T-cell markers, and neutrophil markers in BRCA (Figures 4A–C). Similarly, B-cell markers, macrophages markers, and dendric cells markers were also correlated with high expression of TMPRSS2 in LUAD (Figures 4D–F). These results strongly confirmed the positive correlation between TMPRSS2 and immune cell infiltration in LUAD and BRCA.

In order to explore the prognosis of TMPRSS2 expression levels in different cancers based on immune cells, we analyzed the Kaplan-Meier plotter database and found that high expression of TMPRSS2 in breast cancer had a poor prognosis in enriched CD4^+^ T cells and enriched CD8^+^ T cells (Figures 5A, C), and high expression of TMPRSS2 in LUAD has a better prognosis in enriched B cells, enriched macrophage cells, and enriched CD4^+^ T cells, respectively (Figures 5E, G, I). Interestingly, the prognosis of LUAD and breast cancer affected by the TMPRSS2 expression is consistent with the respective prognosis based on immune cells. These results indicated that the prognosis of LUAD and breast cancer affected by the high expression of TMPRSS2 may be partially influenced by the immune infiltrating cells.

The role of TMPRSS2 in COVID-19 and prostate cancer is widely discovered, but in other cancers is rarely reported. TMPRSS2 is highly expressed in the prostate and is regulated by androgenic ligands and the androgen receptor (Lin et al., 1999). In prostate cancer, TMPRSS2 activated pro-hepatocyte growth factor (HGF) and then promoted c-MET receptor tyrosine kinase signaling to regulate cell carcinogenesis (Lucas et al., 2014). c-MET is a receptor tyrosine kinase abnormally activated in many cancer types, such as renal, liver, head and neck, gastroesophageal, breast, and lung cancer (Reis et al., 2018), and activates several intracellular signaling pathways including RAS-MAPK, PI3K-AKT, RAC1, and PAK to promote the progression of cancers (Graveel et al., 2013). Moreover, HGF/c-MET signaling is also involved in the regulation of tumor microenvironment, immune infiltration, and immune response (Hartmann et al., 2016; Reis et al., 2018; Chen et al., 2019; Li et al., 2019; Zambelli et al., 2021). Thus, TMPRSS2- HGF/c-MET axis could be a potential mechanism that participated in the correlation of TMPRSS2 expression with immune infiltration and prognosis in LUAD and BRCA. In this study, we demonstrated that TMPRSS2 affects the prognosis of LUAD and BRCA through tumor immune cell infiltration, and the biological mechanism of TRPRSS2 needed to be in-depth experimental exploration in the future.

## Data Availability

The original contributions presented in the study are included in the article/Supplementary Material, further inquiries can be directed to the corresponding authors.
