# Supplementary material for: TMPRSS2 Serves as a Prognostic Biomarker and Correlated With Immune Infiltrates in Breast Invasive Cancer and Lung Adenocarcinoma
Source: Front Mol Biosci. 2022 Apr 26;9:647826. doi: 10.3389/fmolb.2022.647826 (PMC9086397; doi:10.3389/fmolb.2022.647826)
Supplement: Supplementary file 1 [file DataSheet1.docx]

**TMPRSS2 serves as a prognostic biomarker and correlated with immune infiltrates in breast invasive cancer and lung adenocarcinoma**

Xinhua Xiao^1†*^, Huizhuang Shan^2†^, Yangyang Niu ^3†^, Peihong Wang^4^, Donghe Li^4^, Yuyin Zhang^4^, Jiayi Wang^1^, Yingli Wu^5^ and Hua Jiang^1*^

^1^ Department of Hematology/Oncology, Guangzhou Women and Children's Medical Center, Guangzhou, China.

^2^ Laboratory Medicine, Guangdong Provincial People’s Hospital, Guangdong Academy of Medical Sciences, Guangzhou, Guangdong 510000, China.

^3^ Department of Nephrology, Shanghai Tongji Hospital, Tongji University School of Medicine, Shanghai, China.

^4^ State Key Laboratory of Medical Genomics, Shanghai Institute of Hematology, Collaborative Innovation Center of Hematology, National Research Center for translational Medicine, Ruijin Hospital, Shanghai Jiao Tong University School of Medicine, Shanghai, China.

^5^ Hongqiao International Institute of Medicine, Shanghai Tong ren Hospital/Faculty of Basic Medicine, Chemical Biology Division of Shanghai Universities E-Institutes, Key Laboratory of Cell Differentiation and Apoptosis of the Chinese Ministry of Education, Shanghai Jiao Tong University School of Medicine, Shanghai, 200025 China.

^†^ These authors contributed equally to the work.

^*^ Corresponding Authors: Hua Jiang (jiang_hua18@sina.cn)

Xinhua Xiao (xinhxiao@163.com)

**Supplemental materials and methods**

**Cell lines and reagents**

The human normal breast epithelial cell line MCF10A and human breast carcinoma cell lines MDA-MB-231, MDA231-LM2, MDA-MB-468 and MCF7 were provided by Huizhuang Shan. Human normal lung epithelium cell line BEAS-2B and lung adenocarcinoma cell lines A549, H1975 and H1666 were obtained from Yangyang Niu. The above cell lines were cultured in DMEM (Gibco, Grand Island, NY, USA) supplemented with 10% fetal bovine serum (Gibco, Grand Island, NY, USA).

**Real time quantitative RT-PCR**

According to the manufacturer’s instructions, total RNA of the cell lines, 11 diagnosed LUAD patients’ tumor tissues and adjacent normal tissues (Shanghai Tongji Hospital) was extracted by TRIzol reagent (Invitrogen, Carlsbad, CA), and then the above RNA was transcribed into complementary DNA separately utilizing PrimeScript RT reagent kit (Takara, Shiga, Japan). Subsequently, real-time PCR was performed. The ΔΔCt method was used to evaluate gene expression. The primers used in study were as follows: TMPRSS2-QF1 sense: 5′ CAAGTGCTCCAACTCTGGGAT- 3′, antisense: 5′-AACACACCGATTCTCGTCCTC-3′; GAPDH sense: 5′-GGACCTGACCTGCCGTCTAG-3′, antisense: 5′ -GTAGCCCAGGATGCCCTTGA-3′. Each experiment was done in triplicate.

**Supplementary Table 1. Relationships between TMPRSS2 expressions and prognoses in different cancers in PrognoScan database.**

| **Dataset** | **Cancer type** | **Subtype** | **Eenpoint** | **N** | **HR [95% CI-low CI-upp]** | **COX P-Value** |
| --- | --- | --- | --- | --- | --- | --- |
| GSE3143 | Breast cancer | - | Overall Survival | 158 | 0.89 [0.57 - 1.38] | 0.591432 |
| GSE12276 | Breast cancer | - | Relapse Free Survival | 204 | 1.10 [1.00 - 1.21] | 0.0479541 |
| GSE6532-GPL570 | Breast cancer | - | Relapse Free Survival | 87 | 13.96 [1.54 - 126.26] | 0.0189748 |
| GSE9195 | Breast cancer | - | Relapse Free Survival | 77 | 0.79 [0.05 - 12.66] | 0.867959 |
| GSE9893 | Breast cancer | - | Overall Survival | 155 | 1.48 [1.09 - 1.99] | 0.0108505 |
| E-TABM-158 | Breast cancer | - | Overall Survival | 117 | 0.83 [0.40 - 1.76] | 0.632818 |
| GSE12945 | Colorectal cancer | - | Overall Survival | 62 | 0.22 [0.01 - 8.76] | 0.420054 |
| GSE17536 | Colorectal cancer | - | Overall Survival | 177 | 0.75 [0.51 - 1.09] | 0.128135 |
| GSE17537 | Colorectal cancer | - | Overall Survival | 55 | 0.76 [0.33 - 1.72] | 0.507041 |
| GSE22138 | Eye cancer | Uveal melanoma | Distant Metastasis Free Survival | 63 | 1.95 [0.14 - 27.33] | 0.621002 |
| GSE2837 | Head and neck cancer | Squamous cell carcinoma | Relapse Free Survival | 28 | 0.01 [0.00 - 17.82] | 0.243781 |
| jacob-00182-MSK | Lung cancer | Adenocarcinoma | Overall Survival | 104 | 0.35 [0.13 - 0.94] | 0.0362315 |
| GSE13213 | Lung cancer | Adenocarcinoma | Overall Survival | 117 | 0.68 [0.51 - 0.89] | 0.00614575 |
| GSE31210 | Lung cancer | Adenocarcinoma | Overall Survival | 204 | 0.59 [0.41 - 0.86] | 0.00581929 |
| GSE31210 | Lung cancer | Adenocarcinoma | Overall Survival | 204 | 0.58 [0.44 - 0.76] | 0.000115681 |
| GSE3141 | Lung cancer | NSCLC | Overall Survival | 111 | 1.08 [0.87 - 1.34] | 0.476362 |
| GSE14814 | Lung cancer | NSCLC | Overall Survival | 90 | 0.89 [0.58 - 1.38] | 0.613366 |
| GSE17710 | Lung cancer | Squamous cell carcinoma | Overall Survival | 56 | 0.84 [0.63 - 1.11] | 0.223626 |
| GSE9891 | Ovarian cancer | - | Overall Survival | 278 | 0.78 [0.57 - 1.06] | 0.11693 |
| GSE9891 | Ovarian cancer | - | Overall Survival | 278 | 0.37 [0.17 - 0.80] | 0.0123117 |
| DUKE-OC | Ovarian cancer | - | Overall Survival | 133 | 5.41 [1.68 - 17.48] | 0.00473586 |
| DUKE-OC | Ovarian cancer | - | Overall Survival | 133 | 0.46 [0.14 - 1.47] | 0.190217 |
| GSE8841 | Ovarian cancer | - | Overall Survival | 81 | 0.79 [0.44 - 1.40] | 0.417528 |
| GSE26712 | Ovarian cancer | - | Overall Survival | 185 | 1.78 [1.03 - 3.07] | 0.0393553 |
| GSE17260 | Ovarian cancer | - | Overall Survival | 110 | 0.82 [0.55 - 1.24] | 0.357011 |
| GSE14764 | Ovarian cancer | - | Overall Survival | 80 | 1.09 [0.81 - 1.46] | 0.581942 |
| GSE19234 | Skin cancer | Melanoma | Overall Survival | 38 | 2.21 [0.62 - 7.85] | 0.218488 |

**
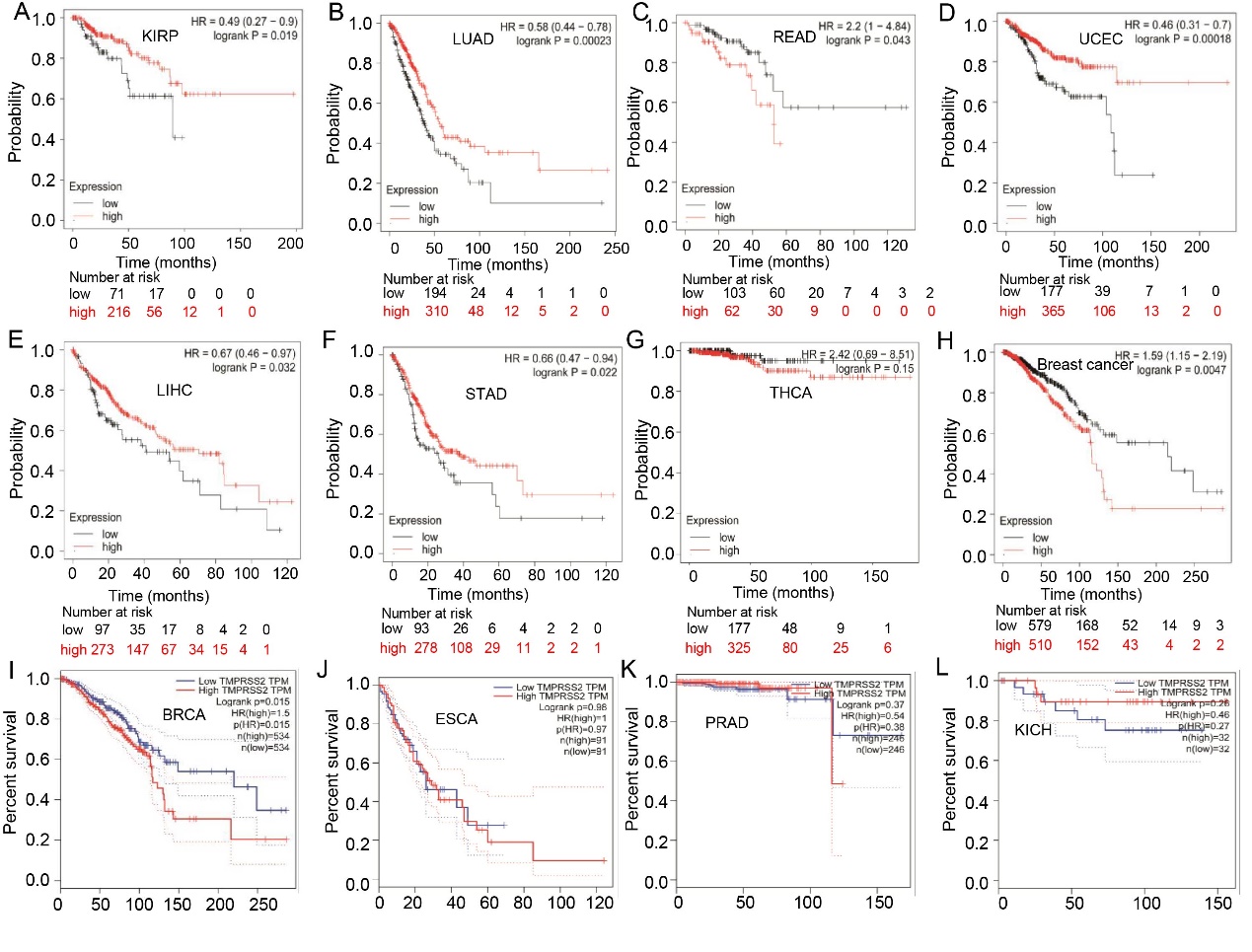
**

**Supplementary Figure 1. The prognosis of high and low TMPRSS2 expression in various types of cancer.** Overall survival comparing the high and low expression of TMPRSS2 in kidney renal papillary cell carcinoma (KIRP) (A), lung adenocarcinoma (LUAD) (B), rectum adenocarcinoma (READ) (C), uterine corpus endometrial carcinoma (UCEC) (D), liver hepatocellular carcinoma (LIHC) (E), stomach adenocarcinoma (STAD) (F), thyroid carcinoma (THCA) (G) and Breast cancer (H) were analyzed by Kaplan-Meier plotter database. Overall survival comparing the high and low expression of TMPRSS2 in breast invasive carcinoma (BRCA) (I), esophageal carcinoma (ESCA) (J), prostate adenocarcinoma (PRAD) (K) and kidney chromophobe (KICH) (L) were analyzed by GEPIA database.

**
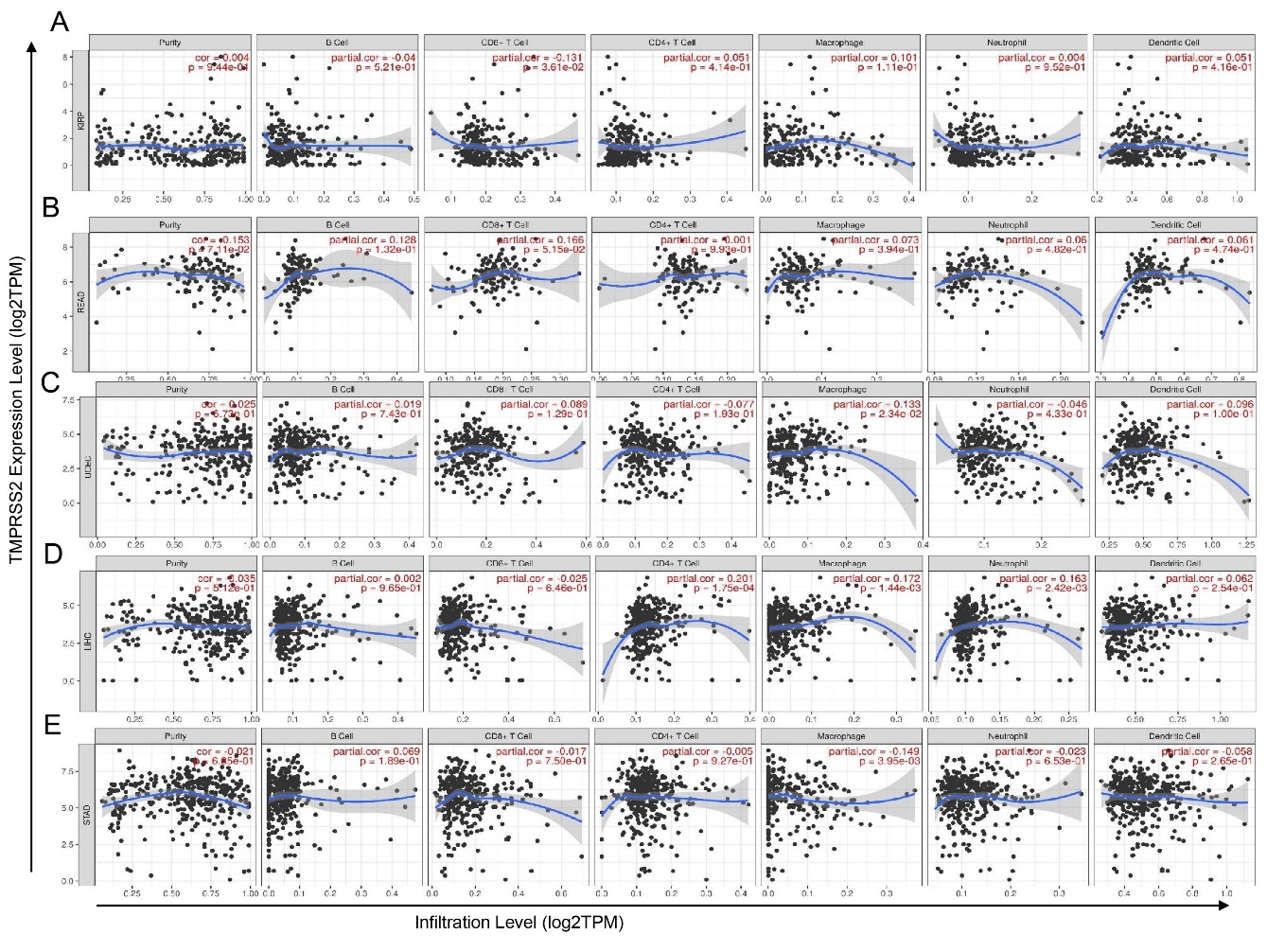
**

**Supplementary Figure 2.** **Correlation of TMPRSS2 expression with immune cells infiltration levels in various cancer.** Correlation of TMPRSS2 expression with immune cells infiltration levels in kidney renal papillary cell carcinoma (KIRP) (A), rectum adenocarcinoma (READ) (B), uterine corpus endometrial carcinoma (UCEC) (C), liver hepatocellular carcinoma (LIHC) (D) and stomach adenocarcinoma (STAD) (E).

**
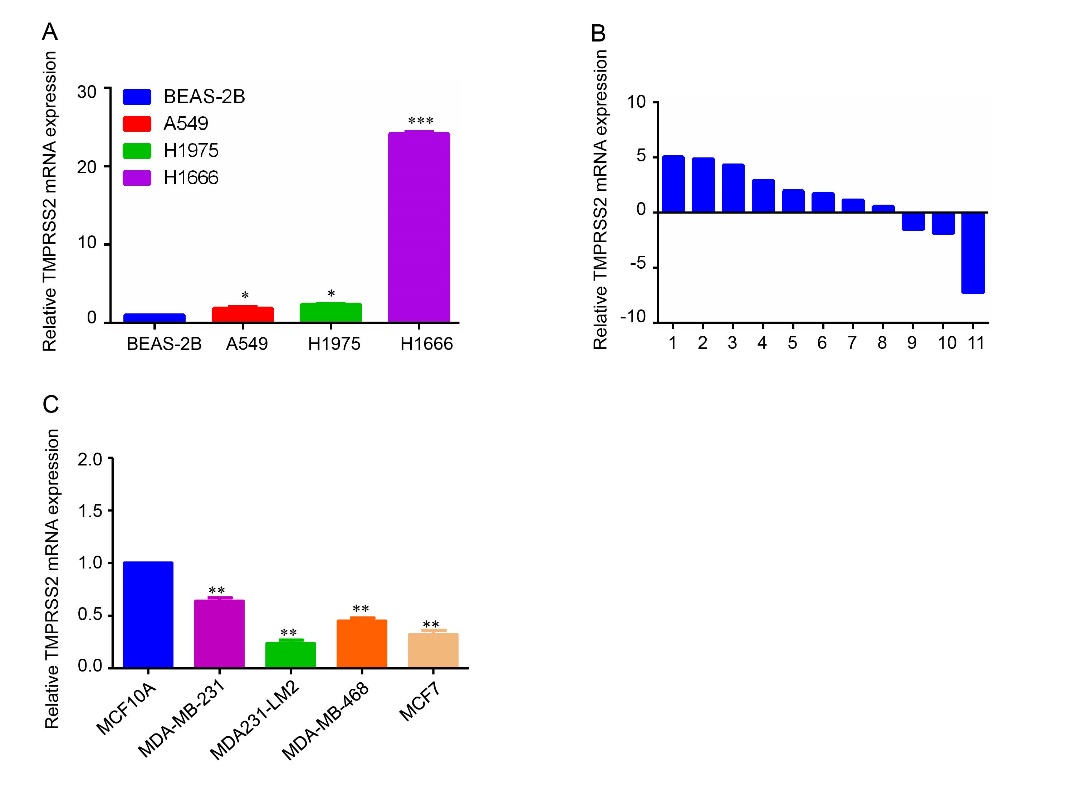
**

**Supplementary Figure 3.** **TMPRSS2 expression in lung adenocarcinoma and breast invasive cancer.** (A) The relative mRNA expression of TMPRSS2 in human normal lung epithelium cell line BEAS-2B and LUAD cell lines A549, H1975 and H1666 were detected by qRT-PCR. (B) TMPRSS2 mRNA expression in 11 diagnosed LUAD patients’ tumor tissues compared with adjacent normal tissues were analyzed by qRT-PCR. (C) The relative mRNA expression of TMPRSS2 in human normal breast epithelial cell line MCF10A and BRCA cell lines MDA-MB-231, MDA231-LM2, MDA-MB-468 and MCF7 were analyzed by qRT-PCR. All values represent the means ± S.D. of three independent experiments. **P* < 0.05, ***P* < 0.01, ****P* < 0.001 versus the control.
